# Biomimetic Adaptive Building Façade Modeling for Sustainable Urban Freshwater Ecosystems: Integration of Nature’s Water-Harvesting Strategy into Sun-Breakers

**DOI:** 10.3390/biomimetics9090569

**Published:** 2024-09-19

**Authors:** Berkan Kahvecioğlu, Güneş Mutlu Avinç, Semra Arslan Selçuk

**Affiliations:** 1 Department of Architecture, Faculty of Architecture, Gazi University, 06560 Ankara, Turkey; semraselcuk@gazi.edu.tr; 2 Department of Construction and Technical, Erciyes University, 38280 Kayseri, Turkey; 3 Department of Architecture, Faculty of Engineering and Architecture, Mus Alparslan University, 49250 Mus, Turkey; g.avinc@alparslan.edu.tr

**Keywords:** adaptive building, biomimetic façade modeling, clean water and sanitation, sun-breaker, sustainable cities and communities, water-harvesting, urban freshwater ecosystem

## Abstract

Urban freshwater ecosystems have many critical functions, such as providing water to all living things and supporting biodiversity. Factors such as water pollution, increased water consumption, habitat loss, climate change, and drought threaten the health of urban freshwater ecosystems. Looking for solutions to these challenges, this article aims to recycle water and return it to its life cycle using a climate-sensitive water collection strategy. The model focuses on the biomimetic method as a basic strategy. In this regard, the concept of water-harvesting has been examined in detail by conducting a deep literature review, including architecture and engineering disciplines. With all these data obtained, a synthesis/integration study was carried out by developing a model proposal based on adaptive building façade elements to solve the water problems experienced in cities. The model proposal, which is directly related to the titles of “Clean Water and Sanitation (SDG 6)” and “Sustainable Cities and Communities (SDG 11)”, which are among the Sustainable Development Goals (SDGs), aims to provide different perspectives on the disciplines with its superficial and functional features. In this context, it is anticipated that the article will become an indispensable resource for other researchers working on the subject.

## 1. Introduction

The world’s future depends on the cooperation of scientists, researchers, policymakers, practitioners, and controllers from different disciplines and the development of more sustainable living standards and strategies. For example, with the Annual Environment Policy Review reports organized every year with the initiatives of the European Commission, the current environmental developments at the European Union and national levels are monitored, and future risks and potentials are discussed together. As stated in the report prepared by the European Commission in 2009, the increasing environmental pressures indicate the need for innovative alternatives to the ecosystem-destroying technologies of today’s industrial age; a solution [1]. So much so, that it is stated that approximately 7 trillion US dollars are invested annually by both the public and private sectors against activities that damage the ecosystem globally [2]; this number is more critical when considering interrelated crises such as climate change, biodiversity loss, damage to aquatic ecosystems, and land degradation.

The combination of many problems, such as rapid urbanization on a worldwide scale, disasters, climate change, and resource consumption, poses severe challenges for 21st-century cities. Along with urban pressures, especially the rapidly changing demographic structure of megacities, increasing transportation problems, resource depletion, and various natural phenomena (flooding, overheating, etc.) occurring frequently, the urban freshwater ecosystem, along with biodiversity, is being damaged, and alternative solutions are being sought [3,4,5,6,7,8,9]. According to the United Nations, approximately half of the world’s population lives in urban areas [10], and this proportion is projected to increase to approximately 70% by 2050 [11]. This also emphasizes the need for severe measures to be taken. Therefore, it is clearly understood that living standards in cities will be more complex, and all disciplines should create different solutions regarding the environment and protecting clean water ecosystems.

Arid and semi-arid belts, which arise due to the uncontrolled consumption of water in most of the world, irregular rainfall, and the loss of most of the valuable water as surface runoff in a short period, also bring serious risks globally [4,9,12,13]. Therefore, recent water scarcity, increased consumption, and a decrease in rain put nature and humanity at significant risk [4,6,8,9,12,14,15]. Against these water-related problems, this study considered the water-harvesting strategy developed by living things in nature as a critical resource, with the reference that science receives from nature. Collecting and re-evaluating the flow of water without causing erosion constitutes one of the most essential objectives of sustainability [16,17]. The concept of biomimicry is a nature-inspired approach. It is considered a response to the increasing demands to find alternatives to the ecologically destructive technologies, systems, and methods of the current industrial age, which are expected to solve today’s unsustainable human–nature relationship [18]. This strategy and approach can enable ecosystems and built environments to adapt to climate change by mimicking nature through durable, sustainable, and adaptable natural regeneration [19]. In this respect, the adaptive reuse of buildings is essential for sustainability and the future [20].

There are many new and different approaches to addressing global environmental problems in various disciplines. For example, risks such as climate change and damage to the ozone layer have brought the concept of energy to the forefront, and engineering and architectural disciplines have also brought the subject under the spotlight [21,22,23,24]. Shading systems and sun-breakers have gained popularity, especially considering strategies related to façades, and they have been ensured to control the energy used in buildings [23,24]. From this point of view, this study offers an alternative perspective in addition to the energy-efficient façade designs of sun-breaker building elements from shading systems, with an approach that includes architectural and engineering disciplines in perspective. At the same time, the importance of the study is revealed by the fact that the field is given a new dimension apart from the focused perspective and that the water-harvesting strategy, considered and determined to be necessary, contributes to the freshwater ecosystem in urban areas.

Being inspired by nature is an essential subject within architecture, like other disciplines. Various biomimetic design strategies have been developed, and attempts have been made to ensure the integration of biomimetics with architecture [25,26,27,28]. However, these integration initiatives still face difficulties [29,30,31]. Future studies are expected to provide perspectives and contributions if these difficulties are overcome. From this point of view, this study contributes to the literature by revealing the development and deciphering potential of the existing system; the subject is considered and approached in the context of engineering, architecture, climate adaptability, and the sustainability of the connections between urban and ecological systems. This article presents a system initiative, with perspectives emphasizing the importance of an interdisciplinary approach to urban freshwater ecosystems and the achievements obtained from joint analyses.

## 2. Materials and Methods

This study, which deals with an integration initiative, aims to provide an architectural solution based on the main problems of global environmental issues such as drought, desertification, water scarcity, and water loss. The model’s integration initiative and development process started with a deep literature review. After the data collection and analysis processes, thematic analysis was used to categorize and explore the development stages of the model. From this point of view, the concept of biomimicry was first considered, and the current studies in the literature were evaluated. After that, within the architecture discipline’s framework, a literature review was conducted, primarily on water harvesting/acquisition; studies and approaches in the field were put forward. The literature about sun-breakers, which are taken as the main design element of this study and can be preferred as aesthetic building elements, is presented alongside their functional architectural value in buildings. The focus of both research topics on the literature on a global scale has provided critical perspectives and references in terms of opening horizons for the study. This study has formed an essential basis for the development of the model. The bio-sun-breaker model, designed as a result of the development stages, proposes a different integration initiative that can solve water loss problems with nature’s strategic water-harvesting approach and contribute to urban freshwater ecosystems along with functional and aesthetic purposes in the design of buildings.

**Figure 1:**
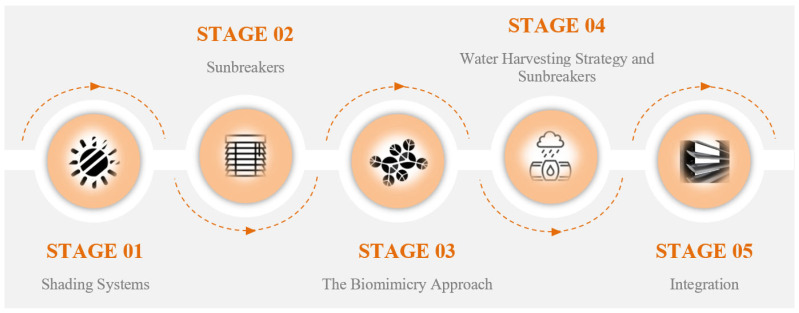
Model proposal development diagram.

The literature review was considered to have two stages. As a strategy, the approaches to water-harvesting and sun-breaker building elements from the selected shading systems are discussed in detail. The points that can improve the model proposal are indicated more in the analysis and results. In this context, the research and studies conducted on sun-breaker structural elements in the literature have been examined in detail, and gaps in the field have been identified. After that, the structural component of the sun-breaker was re-examined through the concept of biomimicry, which is the primary approach of this study, and alternatives that can be solutions to problems were developed by presenting perspectives. With the biomimicry approach, sun-breakers have been integrated with the water-harvesting strategy, and a new model proposal has been introduced to the field (Figure 1).

## 3. Literature Review and Theoretical Background

The nature-inspired/learning/application approach, defined by concepts such as biomimicry, biomimetics, bio-knowledgeable, biomimesis, bio-imitation, and bio-inspired, constitutes the primary approach of this study because it offers different perspectives and prioritizes sustainability. Biomimicry (bio—life, and mimesis—to imitate) is a design approach inspired by the functional concepts of an organism or a natural ecosystem [32]. According to Benyus [33], biomimicry imitates the processes occurring in nature to create innovative solutions with sustainable design. Bio-imitation generally refers to using ecological criteria to evaluate sustainability by addressing it and to create models and designs inspired by nature within the framework of forms, processes, and ecosystems [33,34]. It has been observed in the literature review that the biomimicry approach is an interdisciplinary research field in which experts from different backgrounds, such as architecture, education, health, philosophy, computer science, biomedicine, physics, and chemistry, are involved in biology and engineering to create highly sustainable products [35,36,37,38].

The biomimicry approach offers inspiration to disciplines in three ways: organism (the imitation of nature), behavior (the imitation of natural processes), and ecosystem levels (the imitation of ecosystem functioning principles) [39]. At the organism level, design and architecture come to the foreground, and inspiration is taken from the shape of a building, the form of the design, or the structure of the model. At the behavioral level, the unity and interaction between the ecosystem and its environment inspire its design. At the ecosystem level, the main focus is on the overall holistic relationships with other parts of the organism, and how they interact with each other is of interest. Each stage constitutes an essential approach in terms of its characteristics, the fact that it can be interpreted in many disciplines, and the fact that it provides broad perspectives.

Biomimicry against rapidly occurring climate changes and environmental degradation is critical today. The biomimicry approach, which has recently increased in popularity as a concept, has also been adopted in recent centuries and is inspired by nature. For example, in 1482, Leonardo da Vinci invented a flying machine inspired by the flight mechanisms of birds and bats, and this study is considered the first example of biomimicry [40,41]. Therefore, the need to study and imitate nature to find practical solutions to human needs is not a new strategy, but a process that continues and develops from the past to the present.

The approaches used for biomimicry in different disciplines are broad and involve various solutions. Thus, using nature-inspired strategies provides excellent benefits for each area. For example, although large in volume, a bionic car prototype inspired by the box fish (Ostracon Meleagris) has a different vehicle design, with a small wheelbase [42]. The bionic car consumes less fuel because the volume is more aerodynamic as a result of the imitation of a box fish. The tree development model is also supported, using the minimum necessary materials in the car’s structure. Therefore, a new perspective has been created in the considered vehicle design, which is more sustainable and includes comfort in a developmental sense. Other examples in different disciplines include hydrophobic and friction-reducing materials in textile production [43,44], velcro inspired by cocklebur seed shells [45], sea current turbines inspired by dryobalanops aromatica seeds [46], self-cleaning paints [47,48], and super-hard ceramics imitating mother-of-pearl pearls [49]. Similarly, there are studies produced using a biomimetic approach.

### 3.1. Water-Harvesting Strategy in Nature

The water-harvesting approach, one of nature’s many strategies, offers one of the solutions to the most critical water problem today. Animals and plants are the choices for water-harvesting-based solutions in the literature because water shortages are becoming increasingly severe globally, as shown in Table 1 and Table 2. It is observed that these studied species exhibit similar approaches in terms of their perspectives and working principles. The approach strategies of the animal and plant species included in the tables have enabled the development of the model proposed in this study and provided a meaningful perspective.

When we look at the plants and animals that include the water-harvesting strategy in the tables, it is seen that their capacity to adapt to nature is quite strong. In addition, the fact that similar plant and animal species are often found in studies that address water-harvesting strategies indicates that species in nature have identical strategies. Table 1 and Table 2, which summarize the sensitive cycle of nature in this area of water acquisition, are of critical value in structural and operational planning and are expected to reference other disciplines to be studied in the future.

When the animal and plant species in the tables are examined, it is seen that they have different structures and characteristics, but they apply similar strategies for the same purpose. When going into detail, it has been determined that the superficial features of plants and animals have an identical form and shape and consist of structures that direct water. Curved and bumpy structures are composed of the complex and soft shells of animals, and paths consist of needles and capillary structures in plants and direct water or moisture in the air by holding them. These morphological and behavioral characteristics have been referenced many times in past studies and have paved the way to the model proposal in this article.

### 3.2. Biomimicry Approach and Studies in Architecture

People resort to innovative methods by taking inspiration from nature to create different solutions to various challenges and maintain their lives. Architecture is also among the disciplines that fortify themselves from nature and often take reference from nature. For this purpose, the discipline of architecture is based on the passive, efficient, and cyclical design principles found in nature by learning from nature to achieve sustainable and conscious architectural design and minimize environmental impact [69]. Biology and architecture are also prerequisites for each other [70].

When we look at bio-inspired projects directly or indirectly related to architecture, systems that take different importance or provide advantages to climatic conditions have been integrated. For this reason, biomimicry has significant potential and benefits in architecture and design. For example, many strategies and problem-solving approaches, models, and projects have been developed, such as the shape and behavior of certain animals that adapt to the effects of the sun and wind; examples of nature approaches in construction materials; and different approach solutions related to heating, cooling, and lighting on building envelopes and façades, inspired by plants due to extreme changes in climatic conditions [37,71,72,73,74].

In architecture, the concept of inspiration from nature is generally seen to be carried out in research and development studies on topics such as light structures, water-harvesting, and thermoregulation. This study, which addresses the water problem, one of the world’s leading climate crises, also analyzed various studies by assessing the current perspectives from the perspective of the architecture discipline. The studies developed as a result of the research and proposed as a model are given in Table 3. When Table 3 is examined, the links between the studies conducted and the proposed studies referencing nature are relatively strong. Models designed with similar and different approaches taken from nature are critical as a reference for subsequent studies. The increase in the quantity and quality of this approach and model studies leads to an unavoidable situation where they have positive effects both in disciplinary and global terms.

The projects in Table 3 show that the building shells or envelopes are inspired by nature and that the water-saving models have progressed to the application stage. When viewed as a whole, although the objectives and working principles of the projects seem to contain similar strategies, it is seen that the approach angles have a horizon-opening effect for future studies. Therefore, developing models with different perspectives regarding approach strategies is critical to the field.

### 3.3. Sun-Breakers

The etymology of sun-breakers is derived from the French language. The word ‘brise’ is expressed as ‘breeze, break and breaking’ in association with the word brise-soleil in the eighth edition of the Dictionary of the French Academy [77], while the word ‘soleil’ corresponds to the word ‘sun’. In English, and within the scope of the literature, it is expressed with the words “sun breaker”, “solar breaker”, or “sunshade”. The architectural element, whose primary function is to break sunlight and control the light coming into the interior, is essential in buildings’ façade style and language.

**Figure 2:**
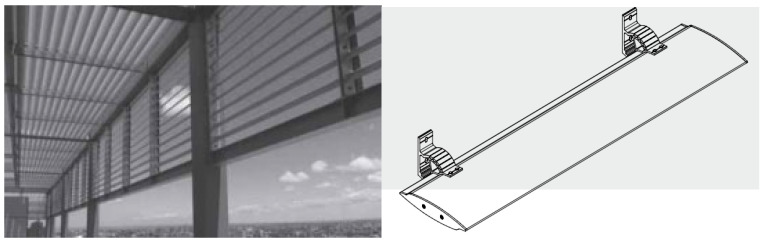
Classic details of the façade sun-breaker.

Shading systems such as sun-breakers, blinds, and curtains (Figure 2) are often used in façades or landscape areas. In addition to its aesthetic value, it has an essential place in architecture and engineering in terms of containing functional importance regarding heat, light, sun, and privacy (the feature of interrupting outdoor and indoor visual communication). According to the literature, the elements found under the title of sun shading and shading systems are expressed with different words in their vocabulary, and other word origins are used together in combinations of concepts [78,79,80,81,82]. It has been found that terms such as sun breakers, sunscreens, sunshades, and blinds include the same, different, or similar systems in shading systems.

**Figure 3:**
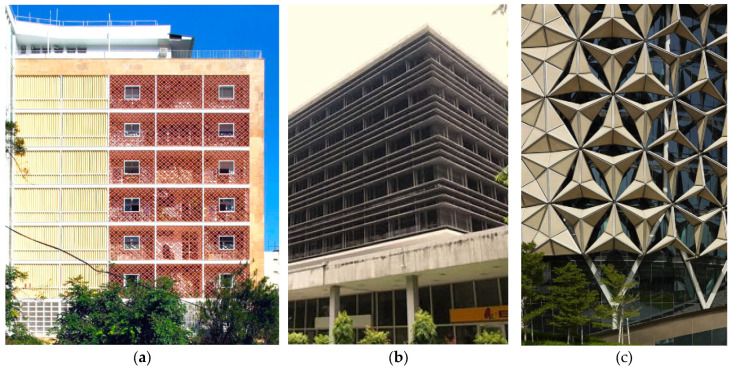
(**a**) Nova Cintra Building, 1948, Brazil [83]; (**b**) The Philamlife Building, 1961, Philippines [84]; (**c**) Al Bahar Towers, 2012, Abu Dhabi [85].

Sun-breakers have been designed in different ways depending on the style of positioning and the material type of the façade. They have added meaning to building façades because of the aesthetic value of the past and present shading systems. They are used in a linear (horizontal/vertical) or grid way with different materials (concrete, cement, wood, metal, aluminum, sheet metal, etc.), preferably in fixed or moving (manual/mechanic) forms. With the development of technology, different forms, purposes, and especially sun-breakers, which are constantly being developed, appear as an indispensable design element for structures today. For example, the sun control system and logic of the sun-breakers in Brazil and the Philippines, which were designed in 1948 (Nova Cintra Building) (Figure 3a) and 1961 (The Philamlife Building) (Figure 3b), as well as on the tower façades built in Abu Dhabi in 2012 (The Al-Bahar Towers 2024) (Figure 3c) as a result of the innovations and opportunities brought by technology with materials, forms, and usage purposes, clearly express the development over the years and changes in perspective approaches.

**Figure 4:**
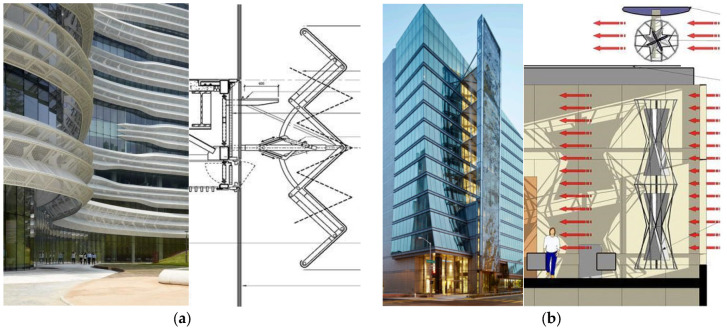
(**a**) CJ R&D Center [86]; (**b**) SFPUC Building [87].

Sun control is of critical importance to minimize the adverse effects of the sun in the discipline of architecture. When we look at the sun- and wind-adapted façade systems, it is seen that the elements that serve as sun-breakers have different behaviors and characteristics. The CJ R&D Center (Yazdani Studio, Gyeonggi-do, South Korea, 2016), which is based on sun control, is one of these examples. A different strategy was developed according to the hole ratios on the panels to adjust the natural light levels of the sun-breaker strips on the façade of the building to the interior (Figure 4a). Another approach, the wind-adapted façade system, was designed to construct the San Francisco Public Utilities Commission Headquarters (SFPUC, KMD Architects and Stevens Architects, San Francisco, ABD, 2012). According to the study results, approximately 7% of the building’s energy needs are met by renewable photovoltaic and wind sources integrated into the building (Figure 4b).

**Figure 5:**
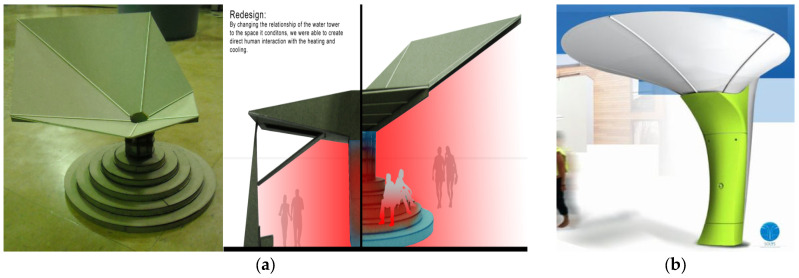
(**a**) Shading systems; (**b**) rainwater-harvesting.

Some studies have investigated shading systems with a rainwater-harvesting strategy. The examples designed with the advantages of rainwater-harvesting and sun-breakers stand out as individual shading systems that generally work independently of the structure. For example, in Figure 5a, a rectangular tower for the water tank and a collection surface were created that also served as a shading tool for heating/cooling the water tank and then created particular conditions for the adjacent area [88]. Another example (Figure 5b) is a sun-breaker system in the form of a concave umbrella made by designer Fabrice Bardon, which can also be used as a fog and rainwater collector. The model is supported by a concave umbrella shape that collects morning mist and rainwater to meet garden watering needs [89].

By including the different word groups identified in the literature review in the study, the latest advancements in global research and the perspectives considered, as well as the sun-breaker elements that are the subject of this research, have been chosen to form the foundation of the review. This literature review provides essential data for future studies to reveal the advantages and disadvantages of sun-breakers and shading systems. Therefore, while shading systems are found in many studies in the literature, the system has been developed using different analysis and research techniques. These studies show that sun-breakers and shading systems approached from various perspectives offer opportunities for other projects and research. The studies that deal with different perspectives are listed as follows:

- Light transmittance and illumination levels [79,90],
- Heat conduction and propagation [79,91],
- Acoustics characteristics [92,93],
- Movement characteristics [80,91,94,95],
- Relative humidity levels [94,95],
- Materials characteristics [79,80,90,91,94,95].

The developments in shading systems show that progress has been made in design and functional terms along with technology, and various practical studies have been carried out. It is understood that this situation will be used more in structures, especially as a result of the sensitivity of the systems to energy efficiency, and it will be in a position open to development. In this study, a new function was integrated into sun-breaker systems, and a model was proposed to develop the system from a different perspective.

## 4. Integration of Water-Harvesting Strategy to Sun-Breakers and Model Proposal

Water acquisition in architectural building shells involves four basic strategies: water collection, water transmission, water transportation, and storage [25]. According to the literature and case study analysis, the backbone of the proposed model and water-harvesting strategy were considered in three steps. For each step, different acquisition strategies in plant and animal species were considered, inspired by nature, and the stages were recorded with developable perspectives. These three steps are shown in Table 4, including the stage, the type of biological organism, and the strategies for handling it.

The strategies selected as biological organisms, and which are a reference for each stage, constitute the critical steps in the development of the model (Table 4). Similar methods for different animal and plant species observed in the literature were influential in developing the stages. So much so, that the first stage, the water collection stage, was based on the spiny structure of the Moloch Horridus animal species, which lives in the desert, has adapted to arid regions, and was integrated into the model. The natural strategies of the lizard species at the point of water acquisition also contributed to other phases of the proposed model (water transmission and water absorption). The natural capillary pathways and asymmetric structures formed by Moloch Horridus’s spiny structure aim to ensure natural circulation at the model’s water flow/transmission point. In the same way, the leaf layout in the water transmission phase constitutes another strategy that is referenced from another aspect of nature. In the leaf layout, like the lizard species, the natural transmission of water is ensured by the different lengths and thicknesses of the asymmetric capillary pathways. It constitutes an important step for storing water with minimal loss. During the water absorption phase, the waters collected on the surface were inspired by stoma, which plant species use effectively for water and air diffusion, and the model was developed together with all other strategies.

### 4.1. Water Collection Stage

**Figure 6:**
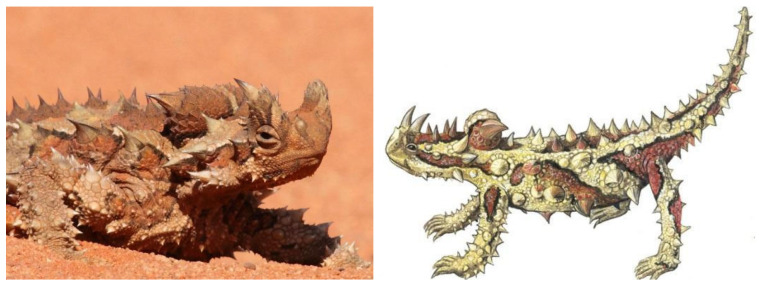
Moloch Horridus.

Water is usually a limiting factor for nature and requires all living things to have special skills to survive in all conditions. For example, it has been found that some frog species have similar passive water collection and water transport capabilities using capillary skins [96]. Again, for lizards that collect moisture, fog, and rainwater, Withers evaluated five possible sources of water intake and considered them in the form of rain, puddles, water droplets caused by dew or fog condensation, moist soil, and thermally facilitated condensation [97]. Within the scope of this study, inspiration was taken from the lizard species “Moloch Horridus,” which is one of the most remarkable animal species in water-harvesting and is known as the “Thorny Devil” (Figure 6).

**Figure 7:**
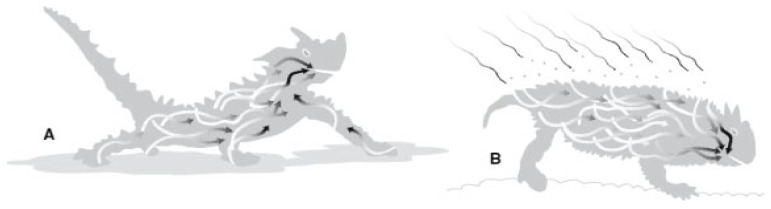
(**A**) Water directed to the mouth in motion; (**B**) water directed to the mouth in a stationary state [100].

The two large horned scales on the thorny devil’s head resemble the figure of a dragon or devil due to the “false head”. This illusion has, therefore, also been influential in its naming. Moloch was used in ancient times for a god often depicted as an ugly monster [98]. The skin of the thorny devil has protruding tips, and each item of water that touches these protrusions helps to collect it in the skin. Water collected through capillary channels between spiny structures is transported to the mouth (Figure 7). Lizard species also collect moisture in deserts by turning the dew condensation at and after extremely low temperatures to an advantage [99].

**Figure 8:**
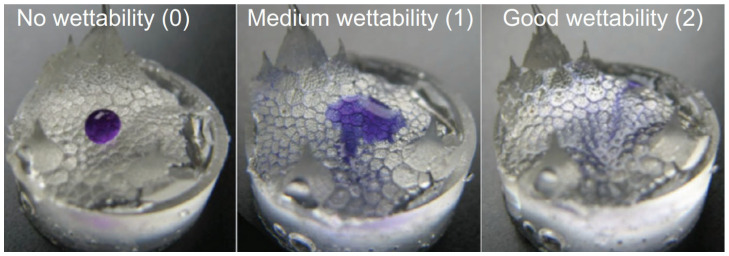
Moloch Horridus skin absorption distribution [101].

Comanns et al. [101] evaluated the water acquisition strategy and skin capillary volumes, detailing he effectiveness of different water sources in filling capillary channels to provide drinking water for the Moloch Horridus (Figure 8). They found that an integrated water collection amount as low as 0.2% of the body weight is sufficient to prewet the skin surface and make the skin super hydrophilic. This situation shows that this ease and strategy for determining surface wettability is expected to be inspired by the skins of the spiny devil species, which can be used to provide a reference for other disciplines to be considered and to encourage the ability to collect water from various sources.

### 4.2. Water Transmission Stage

**Figure 9:**
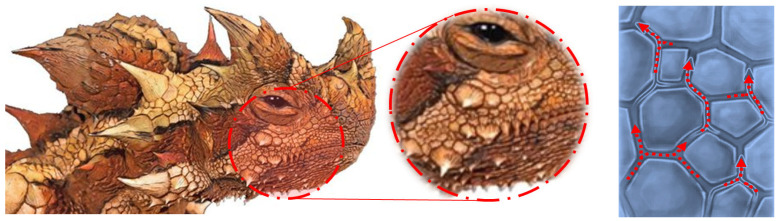
Skin layer (spines and capillary pathways, size change towards the mouth).

The water transmission phase of the model proposal was inspired by the thorny devil lizard of Australia, which is adapted to drought [102]. Water flow is provided to the hexagonal microstructures on the skin surfaces by the hydrophilicity of the skin surface upon contact with trace amounts of water with the skin (pre-wetting). The captured water elements are passively transported by capillary channels in an asymmetric and interconnected system that surrounds the entire body surface of the lizard (Figure 9). When the blisters and channels on the skin surface are directed toward the oral tract, they decrease in size and width, facilitating water flow and accelerating natural flow [60,99,103].

**Figure 10:**
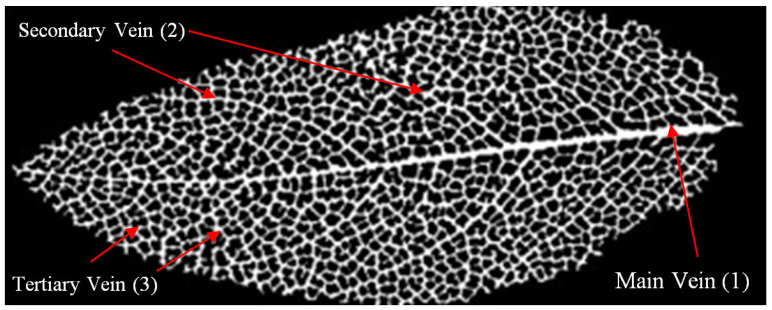
Leaf vein layout [110].

Leaf vein layout, another study strategy for water transmission, was used as another reference for this study. As a transport system, water is distributed to different aspects of the plant through channels in the leaf veins. Generally, the transport system is divided into three degrees in terms of the thickness and length of the vein [104] (Figure 10). Vein number 1 is the main vessel and constitutes the largest channel size. Vein 2 is thinner than the central vein and connects the other two channels. Vein number 3 is the thinnest capillary and constitutes the most extended vascular channel in the carrier system. Changes in vein thickness are the most crucial strategy for allowing water to flow passively. For this reason, many disciplines are bio-inspired by the leaf vein arrangement system. Considering the different perspectives, the leaf vein layout, transport system, and relationships with each other can improve the discipline from a biomimetic point of view [105,106,107,108,109].

### 4.3. Water Absorption Stage

The absorption stage of water constitutes an important step, as does the collection and transportation of water. In nature, there are different strategies for water absorption on the surface. At this point, it is necessary to take the water from the surface before the water from the existing surface is transferred to the stored area. Within the scope of this study, the effective use of stoma by plant species for water and air diffusion was focused on, and inspiration was taken regarding water absorption and air permeability as a strategy.

**Figure 11:**
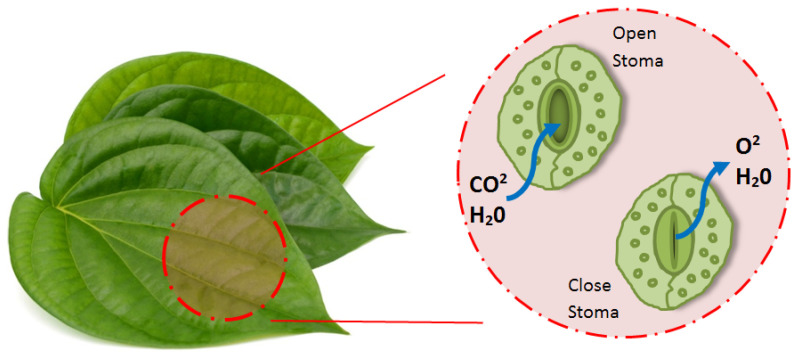
Stoma opening/closure conditions (by the author).

Stomata control the amount of CO_2_ absorbed and the changes associated with water. While many variables affect these processes, stoma features contain significant structural variations depending on the ecological balance or the natural environment [111,112,113,114]. The aperture variability and spatial size of the stoma pores resemble those of two beans, kidneys, or standing coffee beans side by side (Figure 11). The internal pressure of the cell (turgor) determines the shape changes. With increased turgor pressure, the cells bend outwards by stretching in the abdominal membrane, which hardens with increasing pressure. With increasing internal pressure, that is, turgor, the stoma becomes curved, and the stoma opens, and when the turgor decreases, it takes on a closed shape. However, research and discussions are still being conducted on the working mechanics of stomas, and their working principles continue to be analyzed in different ways [114,115,116]. Biomimetic studies have focused on stomata’s transpiration and vapor permeability via this natural strategy [117,118,119,120,121]. Engineering, health, mechanics, and material sciences work on different principles by imitating the system with many other approaches. Model suggestions are seen in many disciplines, and inspiration from nature continues to be used.

## 5. Model Proposal and Working Strategy

**Figure 12:**
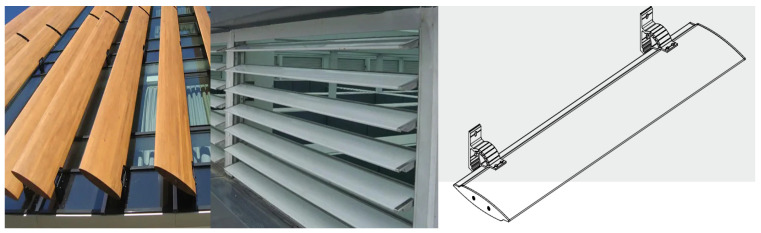
Selected standard sun-breaker.

**Figure 13:**
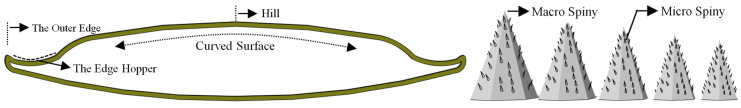
Recommended sun-breaker form and spiny protrusion forms.

A standard model type is often used for the main proposed sun-breaker, and the appropriate production cost has been outlined (Figure 12). Therefore, the proposed stages have a significant advantage in adaptability and applicability to reality. The realization of the design with a foot-on-the-ground approach to the study has added value to the study. For this reason, the proposed sun-breaker model and form characteristics, like those of standard-type sun-breakers, undergo minor changes depending on the water-harvesting strategy. The form changes made, on the other hand, were designed in such a way as to provide an advantage in terms of water collection and accumulation, and the final form was given (Figure 13).

The integration of the strategies used in the animal and plant species studied and inspired by the model has been realized in stages, and the design has been created. During the application of each stage to the model, the proposed layers and strategies were clearly expressed in step-by-step steps. In this context, a form design with areas depending on water collection and water accumulation strategies has been created to determine the sun-breaker form. The water-harvesting strategy, which is the first stage in acquiring water-harvesting for the created form, was inspired by the spiny skin layer of the Moloch Horridus lizard species, and work on the model began. This stage formed the starting point of the surface shape of the sun-breaker model. It aimed to facilitate moisture and fog collection by creating spiny protrusions on the spiny structures (Figure 6 and Figure 9), observed on the skin layer of the thorny devil species, and are the most dominant feature of the water collection strategy (Figure 13). Spiny protrusions were systematically placed on the sun-breaker model in the following stages.

**Figure 14:**
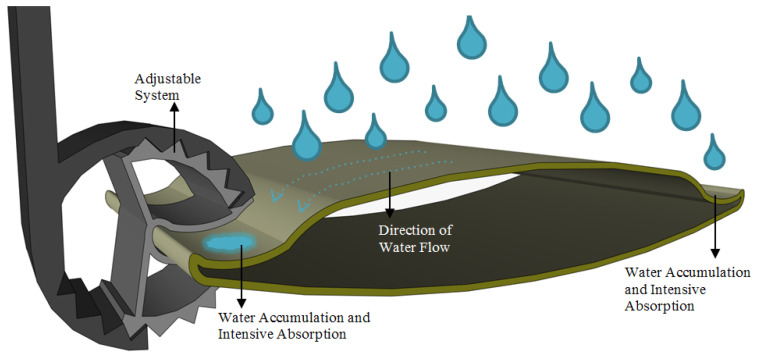
Recommended sun-breaker model and water/moisture/fog intake working principle.

Different strategies were activated during the placement of spiny structures on the sun-breaker model, and systematic formation was achieved. In this direction, the form of the sun-breaker element was first considered during the formation of the sun-breaker surface. As shown in Figure 13 and Figure 14, the design of the form in a curvilinear structure makes an essential contribution to the flow and collection of water. The sun-breaker element hill and the outer edge regions were formed for this design. Water is collected from the air, moisture, and fog, thanks to the spines on the back of the form; the natural flow of water towards the edge cavity is ensured by the shape of the sun-breaker. Moreover, considering the energy-efficient and water-harvesting gain designs of sun-breakers, the proposal of a mobile system has also been included in the model stage. In this context, the aim is to construct a sun-breaker model based on a moving system. Increasing the aesthetic value of the façade also reveals a significant meaning and advantage in sustainable achievements.

**Figure 15:**
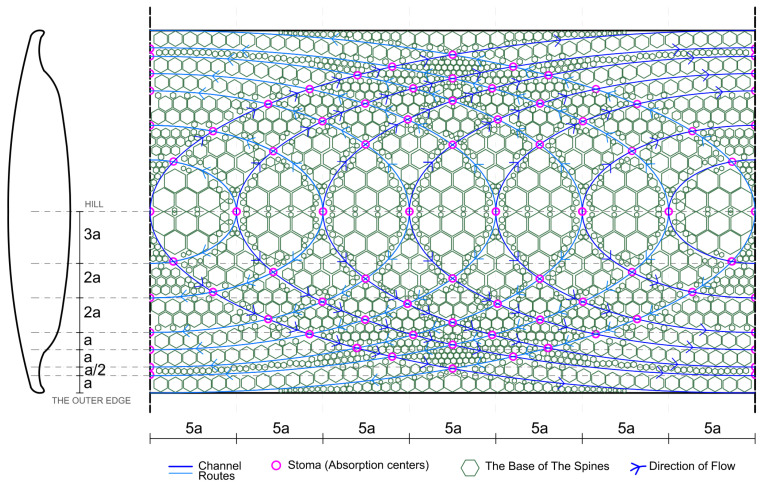
Recommended sun-breaker model: groove, protrusion, and stoma map.

As a result of the conditions imposed by the sun-breaker form, the moment the water from the air falls on the ridge surface or condenses, the flow of water towards the edge is expected to be fast, and the absorption is expected to be low. For this reason, different integrated strategies involving absorption centers, stoma, and channel (groove) formations have been developed. To direct the flow of incoming water grains due to the ridge and curvilinear surfaces brought by the formation of channels, curvilinear forms have been created that slow the water flow rate and facilitate absorption, rather than the perpendicular stripes of channels. Stomata were placed at the intersection points to absorb the water directed with the channels. Due to the low absorption in the back and the high water flow, the number of stomata remained low in the back regions and increased towards the edge cavity regions (Figure 15). Therefore, the possibility of directing the water flow and absorption and controlling the speed has been provided. Studies were completed by placing spiny structures inspired by the lizard species between the created channels and integrating them into the sun-breaker model (Figure 15).

**Figure 16:**
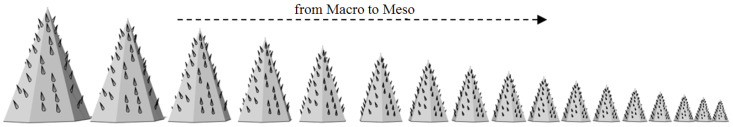
Recommended spiny sizes.

**Figure 17:**
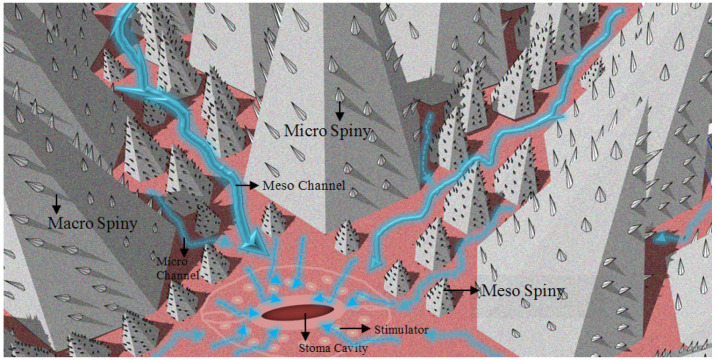
Passive water flows to the stomata.

**Figure 18:**
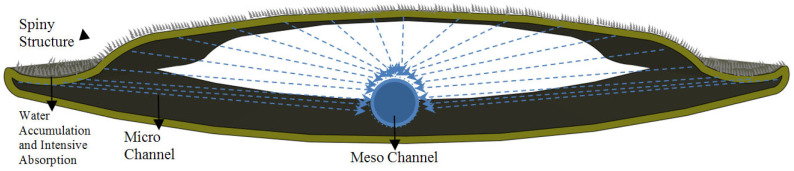
The proposed model perspective section.

Similar strategic approaches in the channels and stomata determined the placement of the spines by taking inspiration from the dimensional changes of the lizard species in the spiny structure moving toward the mouth (Figure 9). In this respect, support is provided for the passive water flow on the model. For this purpose, the spiny structure has been considered in three dimensions: the macro-, meso-, and micro-scales (Figure 16 and Figure 17). Macro-spines constitute the largest spiny structure in the model, as the region in the back area will have the most contact with the air. Meso-spines include spines of different sizes that support macro-spines in a way that describes the route formed by the main channels. Conversely, micro-spines are located on macro- and meso-spines to facilitate water collection and condensation. The dimensional approaches considered were sized and placed depending on the position of the sun-breaker, the desired flow of water, the channel, and the stoma strategies created (Figure 18).

Within the scope of the proposed model, an additional strategy has been proposed that will facilitate the passive flow of water within the channels created on the sun-breaker and help with the water. Considering the thorny devil lizard species (Figure 9) and leaf-vein patterns (Figure 10), this strategy, which will be developed in channel structures, has been confirmed by examples from nature that it will also make an essential contribution to the collection, transmission, and absorption of water. In this context, access to natural pressure and speed will be provided with channel structures, lengths, widths, the attraction of water within itself, and thinning, shrinking, and elongating dimensions. From this point of view, the channels are sized in three ways: macro-, meso-, and micro-channels. Macro-channels are the first and largest channels and cover the channels that directly access the stomata. Meso-channels constitute the channels that are the means for storing water from the stomata. Conversely, micro-channels have been proposed as intermediate channels that provide access to the meso-channel for storing water from capillaries and stomas in the space between spines of different sizes (Figure 17 and Figure 18).

**Figure 19:**
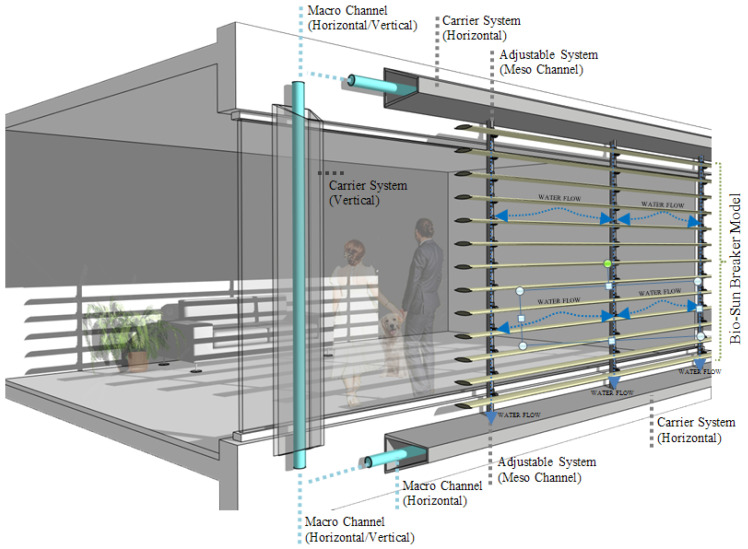
Working principle of the proposed bio-sun-breaker model with a façade.

The strategies recorded in stages and the references taken from nature are integrated into the model. Their relationship to the façade is shown in Figure 19. The diagram clearly shows the working principle and provides water transfer to the greywater storage and distribution center, with horizontal and vertical macro-channels offered as suggestions within the carrier system. At this point, offering alternative water use in buildings with water recycling provides an essential advantage for urban freshwater ecosystems.

The attempt of the model to exhibit a flexible and open-ended approach in terms of design and working principles, along with similar alternatives, can be introduced. This developmental feature also paves the way for the model to be used as an aesthetic building element on the façades of buildings with different architectural designs. In this aspect, the study contributes to the purpose of the proposed model integration and adds critical value. Therefore, this model integration proposal can be repeated and tested together with experimental work processes to ensure the applicability of the design and project initiative and to develop by evolving into different disciplines and dimensions.

## 6. Conclusions

Researchers, designers, policymakers, and investors worldwide are taking essential roles in improving and making sustainable living standards continuous, and different disciplines are trying to develop this issue with other strategies. Therefore, throughout history, designs inspired by nature have taken place in many fields, such as industrial design, health, medicine, materials science, nanotechnology, robotics, and engineering. The discipline of architecture also aims to reach literature and solutions with different perspectives on this subject. Similarly, the results of this study suggest that the discipline of architecture should adopt a method inspired by nature, the biomimicry method, which prevents environmental imbalance, not leaving building designs out of the ecosystem and making them a part of the ecosystem. Understanding and developing this situation, analyzing nature well, identifying its forms, systems, materials, functions, processes, aesthetics, and ecosystems, and discovering relevant solutions and new possibilities for disciplines, are becoming necessary in architecture.

Architectural façade elements play a complementary and additional role in managing the physical environmental conditions of buildings. When an element can control other physical environmental conditions besides the primary construction function, it has an essential advantage in contributing to the sustainability of the building and the environment. For example, daylight in architecture is critical in building design. Volumes with sufficient natural light in the relationship between people and space directly affect the mental and physical health of the user. In addition, considering the impact of buildings on energy consumption, the results also support the sustainability of buildings. However, in addition to these advantages, in this study, in addition to the tasks and characteristics of existing sun-breakers, nature’s water acquisition strategy has been integrated on top of its current potential with a biomimetic approach.

It is envisaged that the inclusion of the proposed model in the project only during the preliminary design of the building to achieve the correct and targeted gains will provide a significant gain. Because of the purpose of use of the building, the shape, the climatic conditions in which the building is located, the size, color, and textural properties of the proposed model are related to criteria such as surface materials and section thickness; it is considered necessary to determine its separation and capacity for each building. The model proposal, which is directly related to the titles of “Clean Water and Sanitation (SDG 6)” and “Sustainable Cities and Communities (SDG 11)”, which are among the Sustainable Development Goals (SDGs), aims to provide different perspectives on the disciplines with its superficial and functional features. In this context, it is anticipated that the article will become an indispensable resource for other researchers working on the subject.

The strategies integrated into the proposed model design constitute the most important privilege of this study to strengthen the urban freshwater ecosystem by providing a different and horizon-broadening perspective. In addition, model integration, which involves the use of a biomimetic approach, is expected to contribute to other biomimetic studies to be conducted in the future with integration methods to structure and space designs that are more sustainable, focused on urban problems in terms of the way they are handled and the model approach. It is necessary to make the subject developable as an essential reference for future research, for studies with other perspective approaches to be physically handled in research and development laboratories, and to complete the study by applying it; performance analyses should be performed. Again, it is necessary to select the building materials for the model’s design, determine the sensitivity conditions at the junction points, test different scales and dimensions, choose the most efficient form and system, and reveal the negative and positive aspects for practical applications. In the same way, it is necessary to integrate the model and determine all the effects related to all economic, commercial, and climatic situations in the application stages to be included in the future-oriented strategies of the model. By conducting experimental studies, breakthroughs can be developed for the future, including other disciplines except for the discipline of architecture, as well as the development of this study and the continuation of its results. Therefore, this article is expected to pave the way for future work by drawing attention to other studies, such as prototype production, simulation tests, and material analysis, with this model initiative.

## Figures and Tables

**Table 1:** Studies and animals inspired by water-harvesting strategy.

Latin Name	Image	Type/ Location	Studies	Bio Inspiration	Latin Name	Image	Type/ Location	Studies	Bio Inspiration
*Crotalus* *cerastes laterorepens*	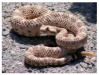	Snake/ Yuma Desert	[50,51]	Rainwater harvesting strategy	*Psammobates tentorius trimeni*	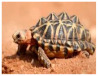	Turtle/SouthAfrica	[52]	Rainwater harvesting strategy
*Physosterna Cribripes/stenocara gracilipes*	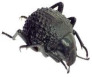	Insect/ Namib Desert	[51,53,54]	The strategy of collecting water droplets	*Onymacris* *unguicularis*	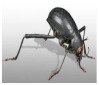	Insect/ Namib Desert	[54,55,56,57]	Upside-down water collection strategy
*Litoria* *caerulea*	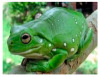	Frog/ Australia	[58]	The skin cavity strategy	*Moloch* *horridus*	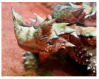	Lizard/ Australia	[51,59,60]	Water collection strategy
*Phrynosoma* *cornutum*	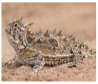	Lizard/ North America	[59]	Water collection strategy	*Uloborus walckenaerius*	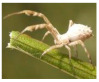	Spider	[51,61]	Spider web strategy

**Table 2:** Studies and plants inspired by water-harvesting strategy.

Latin Name	Image	Type/ Location	Studies	Bio Inspiration	Latin Name	Image	Type/ Location	Studies	Bio Inspiration
*Sekoya sempervirens*	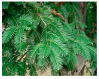	Tree/ California	[62]	Fog collection strategy	*Pseudotsuga menziesii*	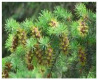	Tree	[62,63]	Fog collection strategy
*Welwitschia mirabilis*	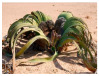	Plant/ Namib Desert	[55]		*Trianthema 8hereroensis (Aizooceae)*	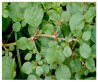	Plant/ Namib Desert	[55,57]	
*Stipagrostis sabulicola*	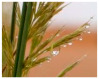	Plant/ Namib Desert	[55,57,64,65]	Water collection strategy	*Opuntia mikrodazisi*	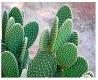	Cactus	[66]	
*Copiapoa haseltoniana*	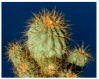	Cactus	[67]	Fog condensation strategy on thorn	*Discocactus horstii*	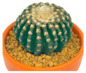	Cactus	[68]	Fog condensation strategy on thorn

**Table 3:** Examples of biomimicry projects and approach strategies.

Project name: Biomimicry Museum [32] Bio-info: Cactus and camel Purpose: canopy design with a bio-inspired strategy as a solution to the water needs of plants. Working Principle: the upper cover of the floor has a large area and is designed inspired by the spongy bone structure of camels in their noses and the spines of cacti that harvest water from the air to reduce evaporation.	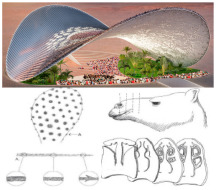
Project name: RainBellows [75] Bio-Inspiration: Ice Plant (mesembryanthemum crystallinum) Purpose: to store rainwater by developing a façade system. Working Principle: it is inspired by the ice flower plant, which can store water. There are façade elements that can increase the storage capacity thanks to the mechanisms that come out of the façade during the rainwater intake of the façade. After the necessary water intake, it is filtered and transmitted to where it is needed in the building.	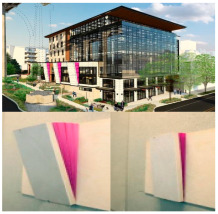
Project name: AquaWeb [76] Bio-Inspiration: honeycombs, spider webs, ice plant, and the mycelium plant. Purpose: a system that can be adapted to the façades of buildings, which harvests and stores atmospheric water with a flexible and modular design and transmits it if necessary.	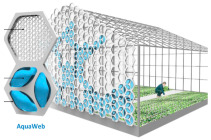
Working Principle: Inspired by honeycombs, there is a web system that can harvest atmospheric water such as rain, fog, and humidity, and imitate the fibers of a spider web. The water that accumulates in the compartments that imitate the sacs of ice plants is stored and transmitted by pipes with the transport feature of mycelium plants.	

**Table 4:** Strategy approach in the model proposal.

Biological Organism	Stage	Strategy
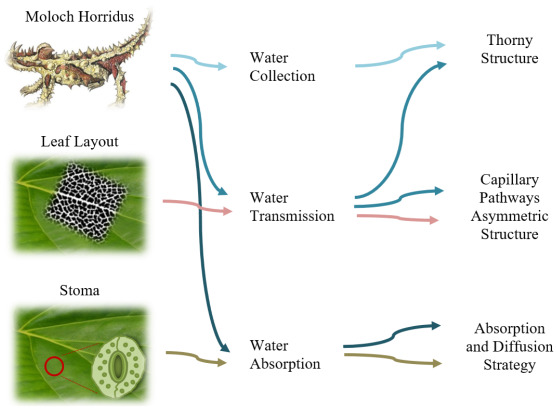		

## Data Availability

Data shall be available from the corresponding author upon reasonable request.
